# Heavy Metal Distribution in Opportunistic Beach Nourishment: A Case Study in Greece

**DOI:** 10.1155/2013/472149

**Published:** 2013-11-28

**Authors:** Spyros Foteinis, Nikolaos G. Kallithrakas-Kontos, Costas Synolakis

**Affiliations:** ^1^Department of Environmental Engineering, Technical University of Crete, 73100 Chania, Greece; ^2^Analytical and Environmental Chemistry Laboratory, Technical University of Crete, 73100 Chania, Greece; ^3^Department of Civil Engineering, University of Southern California, Los Angeles, CA 90089, USA

## Abstract

The existence and distribution of persistent pollutants, such as heavy metals, in coastal sediment used for opportunistic beach nourishment, is a problem that has not received much attention. Here, we assessed the coastal sediments in one restoration project for the occurrence and distribution of heavy metals, by utilizing an Energy Dispersive X-Ray Fluorescence (EDXRF) system. Heavy metal point sources included (i) the effluents of small industries (tanneries), (ii) wastewater treatment plant effluents, and (iii) paint and oil scraps from substandard ship maintenance activities that take place on ports breakwaters. A few neighboring beaches were found to have similar heavy metal concentrations, with mean values of Cu, Zn, and Pb ranging from 80 to 130, 15 to 25, and 25 to 40 mg/kg, respectively. Existing legislation regarding dredging activities in Greece appears insufficient for sustainable and environmentally friendly nourishment. We conclude that before opportunistic beach restoration projects materialize with material borrowed from ports and harbors the quality of the dredged material needs to be assessed.

## 1. Introduction

Beach erosion is claiming many Greek beaches, extensively altered the last decades [1] due to anthropogenic intervention. Until now erosion has not been seriously addressed and thus ecological sensitive ecosystems, like sandy beaches, which also provide shelter to endangered species, may be in danger. Beach erosion in Greece, like the rest of the Mediterranean, can be attributed mainly to human activities, with a common problem that ports and harbors are often situated in beaches which are otherwise in equilibrium. Thus adjacent beaches erode [1]. It is standard practice in developed countries to use dredged material for nourishment [2]. The dredged material can be a major environmental asset, particularly whenever beach sand is available only far offshore. Dredged material is most often used for construction of coastal infrastructure.

When used for opportunistic beach nourishment it should have similar physical characteristics with the beach material, and no persistent pollutants, like heavy metals. To our knowledge and in contrast with other developed countries, Greece has not yet utilized dredging activities for beach nourishment projects nor does it have relevant regulations. The existing legislative framework allows dredged material to be deposited on deep water or to be sold for construction.

Nonetheless, even though opportunistic beach nourishment is a sustainable solution to beach erosion, a major concern is the quality of the dredged material, since seabed sediments from commercial ports and harbors are often heavily polluted [3]. Specifically, heavy metal sediment contamination poses risks to coastal ecosystems and is problematic in dredging activities [4]. Research efforts have focused mainly on the water quality in ports, even though the ecological relevance of sediments is well recognized [5].

Marine sediments are known to act as sinks and reservoirs for pollutants and heavy metals. Heavy metals enter the marine environment through natural processes such as erosion of ore-bearing rocks, wind-blown dust, volcanic activity, and wildfires and through atmospheric and riverine deposition and direct discharges or dumping. Sewage and industrial waste from coastal cities and direct agricultural runoff to the sea add to problem [6]. The high toxicity, nonbiodegradability, and bioaccumulation [7] of such discharges can substantially degrade the host coastlines. Yet, although heavy metals are known to accumulate in organisms living in/on beach sands, the concomitant contamination of beaches has not received adequate attention [8].

Heavy metal accumulation in coastal sediments is quantified through the geoaccumulation index *I*_geo_ [9] as a function of the metal concentration (*C*_metal_) and the natural metal concentration (*C*_metal(control)_):

(1) $$\begin{array}{c} \left(I_{\text{geo}}=\frac{{\text{Log}}_{2}C_{\text{metal}}}{{1.5C}_{\text{metal}(\text{control})\;}}\right). \end{array}$$

While, the occurrence of heavy metals in marine sediments has been thoroughly studied [10–12], there are few reports of heavy metal concentrations in sediments used for beach nourishment [13]. Here, we aim to investigate the occurrence and distribution of heavy metals, such as Cu, Zn, and Pb, in coastal sediments in Chania Prefecture.

## 2. Materials and Methods

### 2.1. Area of Study

**Figure 1:**
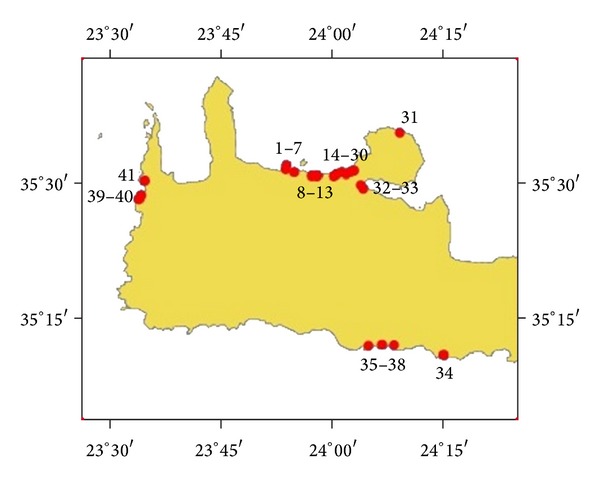
The selected coastal locales.

Western Crete has been rapidly developed in the last three decades for tourism activities. As coastal development in Spain and Italy in earlier decades, substandard engineering design of coastal infrastructure and road building on the shores have caused massive erosion [1]. Our sampling locales were chosen by considering their popularity to visitors, their ecosystems, the existence of possible heavy metals point sources, and whether they were likely to be used as possible sources for opportunistic beach nourishment projects. In total, forty one areas were examined (Figure 1, Table 1) and representative samples were assessed for their heavy metals loads.

### 2.2. Sampling and Analysis

**Figure 2:**
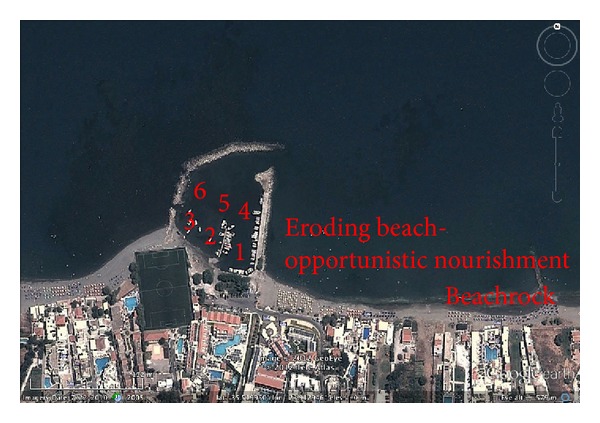
The port's sampling points and the eroding beach of Platanias [15].

Sand samples from the selected 41 areas were collected from November 2009 to March 2010. Samples were extracted from the ground surface with standard 120 mL polypropylene containers, and also at 20 cm below the surface using a sediment corer. Another set was taken in April 2012, from Platanias Port (Figure 2). The latter was chosen because dredged material was to be used for the first ever known opportunistic beach nourishment project in Greece. Six representative sampling points were chosen and surface sediments as well sediments (at 1 m depth) were collected (Figure 2).

Sample homogenization was achieved though drying at 100°C, to remove their water content, and filtered with a 2 mm sieve to remove coarser grain content. Then, 4 g of each homogenized sample was placed on a 32 mm X-ray sampling cup, using Mylar film of 6.0 *μ*m thickness. An Energy Dispersive X-Ray Fluorescence (EDXRF) unit AMETEK Spectro XEPOS benchtop spectrometer was used, with high sensitivity for the entire element range from Na-U, using the X-Lab Pro 4.0 and Turbo Quant Quantification Software. Excitation was through an air-cooled Palladium (Pd) anode X-ray end window tube (40 kV). Measurements were performed with helium gas flushing using a 12-position autosampler. The instrument had three excitation modes, the Compton secondary/molybdenum, the Barkla Scatter/aluminum oxide, and the Bragg crystal/highly oriented pyrolytic graphite (HOPG). Silicon drift detector (SDD), with Peltier cooling and an 8 *μ*m Moxtek Dura-Be window. Its peak to background ratio is 5000 : 1, and the detector resolution 160 eV at 5.9 keV; the irradiation time was 5 min for each excitation mode. The reason that the EDXRF technique was used is due to its capability of directly measuring heavy metals in solid samples with high accuracy, in relative short times, and its multielemental analysis capability [14].

### 2.3. Platanias Opportunistic Beach Nourishment

Platanias beach has undergone extensive erosion during the past decades mainly due to the construction, of the homonymous port on the active beach. The port interfered with the local longshore sediment transport and, as a result, the beach east of the port retreated, while the beach north of the port started to accrete (Figure 2). The port is dredged, once if fills up with sediment. In early 2012, the eastern beach had lost more than its half width compared to its conditions in the 1980s. Several groins (Figure 2) were placed in the 1990s, but did not effectively address the problem, and by now (2013) the groins are detached, year round, and are practically useless.

The last dredging of the port took place in 2004. Since then, it was estimated that the port had accreted about 20,000 m^3^ of sand, having similar geometrical characteristics with the eroding beach. In 2010, the port sediment was identified as a possible source for opportunistic beach nourishment, since the accreted sediment was qualitatively described as having similar heavy metal concentrations with the beach and with background values. Nonetheless to check the safety of the material, thorough heavy metals concentrations reassessment took place in April 2012. It is the first ever, to our knowledge, opportunistic beach nourishment project in Greece.

## 3. Results and Discussion

### 3.1. Heavy Metals Concentrations in Coastal Sediment

**Figure 3:**
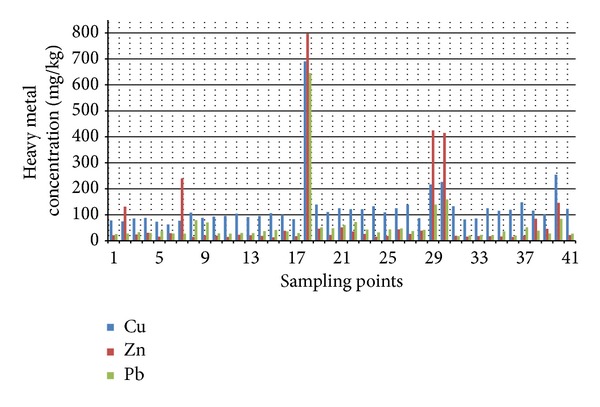
The results of the XRF analysis regarding Copper, Zink and Lead of the selected locales.

The comparative analysis, shown in Figure 3, revealed that the equilibrium beach profiles, as well as examined sediments in river mouths, had a relatively stable and similar concentrations of heavy metals. Only the samples from the Kato Stalos rivulet (samples 8 and 9) had high Pb loads and their sources need to be identified in future studies. Overall, heavy metals loads in coastal sediments of Chania Prefecture were found satisfactory, with reference samples (e.g., beachrock) and their measured values being comparable to the ones measured in a similar coastal environment in Izmit Bay, Turkey [16]. Izmit Bay is located adjacent to the Sea of Marmara, and is a popular destination, which has also extensively modified during the last decades by human activities. Specifically, Cu, Zn, and Pb, were found to have and they significant different values from background, are presented in Figure 3.

Copper had a mean distribution of about 80 to 130 mg/kg in beach sand, and Cu natural loads were found to be higher than Izmit Bay, where Cu ranged from 20 to 77 mg/kg [16]. Zinc natural loads were found to have a very stable spatial distribution, with a mean concentration of about 15 to 25 mg/kg in beach sand; in Izmit Bay, concentrations ranged from 25 to 159 mg/kg [16]. Finally, Pb natural loads exhibited a mean spatial distribution of about 20 to 30 mg/kg in beach sand, similar to the 20 to 43 mg/kg measured in Izmit Bay [16].

Heavy metal point sources were identified and include the following.

**Figure 4:**
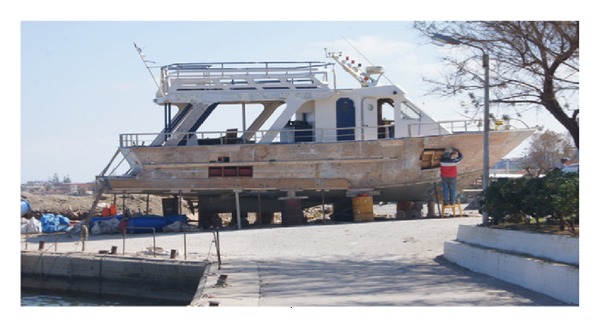
Boat maintenance and repainting, Platanias port, March 19, 2012.

**Figure 5:**
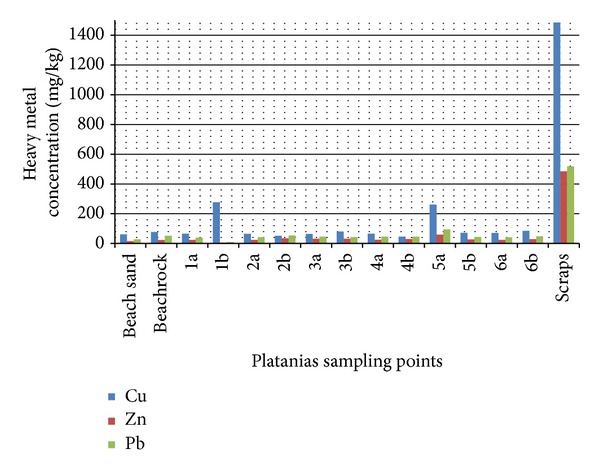
The results of the XRF analysis regarding Copper, Zink, and Lead of Platanias beach and port.

- Effluents from small industries, such as local tanneries situated in Agia Kyriaki, east of Chania, where the homogenized sand sample (sample no 29) as well as the sample from the local port (sample no 30) yielded high heavy metal concentrations (Figure 3): the presence of heavy metals in the sediments can be attributed to pigments and dyes used in tanneries, and to the fact that the wastewaters from this process end up in the sea without treatment.
- Waste water treatment plant (WWTP) effluents: the municipal waste water treatment plant of Platanias discharges about 500 m offshore and in ~12 m depth. Homogenized surface sediment (sample no. 1) collected in front of the WWTP offshore exit did not yield high loads of heavy metals. Nevertheless, homogenized sediment from 20 cm sediment depth in front of the WWTP discharge location (sampling point no. 2) exhibited high Zinc concentration. Zinc is used as an additive in personal care products, such as toothpastes and cosmetics, and ends up in WWTPs, where a part of it settles and bounds in the sludge, and another part is carried away with treated effluents. The low Zn concentration in the surface sediment revealed that there was an active sediment transport at the time of sampling, making heavy metals detection a difficult task.
- Substandard ship maintenance activities, which take place in the Platanias port and along its breakwater: the analysis of homogenized sediment from port breakwaters revealed an order of magnitude higher heavy metal concentration, compared to adjacent beaches, which can be attributed to ship and boat repainting activities. As it can be seen in Figure 4, during repainting activities sometimes no precautionary measures are taken, and hence dyes, pigments, other chemicals, and painting scraps, which contain Zn, Cu, and Pb, end up in the adjacent areas. Both examined breakwaters, that of Nea Chora (sample 18) and of Platanias (Figure 5, scraps) ports, where ship maintenance activities take place (Figure 4); they both contained high loads of heavy metals. For example, sediment from Nea Chora port breakwater had about 7 times higher Cu, 40 times higher Zn and 20 times higher Pb concentration, than their respective natural loads.

Another five small ports examined did not exhibit relatively high heavy metal loads. This indicates that water circulation and sediment transport yielded a significant effect on the occurrence of heavy metal within the borders of provincial ports and harbors.

### 3.2. Opportunistic Beach Nourishment of Platanias Beach

Our results were disseminated to stakeholders and local authorities.

Before nourishment took place, a thorough spatial analysis for heavy metals loads was conducted and samples from the adjacent area as well as from inside of the port were analyzed (Figure 2). During the sampling time, a boat was repainted (Figure 4) and a homogenized collected sample from the breakwater confirmed (Figure 5, sample scraps) that repainting activities excrete heavy metals in port areas and on adjacent beaches. Moreover, one surface sediment sample (Figure 5, sample 5a) and one deeper (1m depth) (Figure 5, sample 1b) in the borrowed material were enriched with Cu, while the other samples had heavy medal concentrations similar to the adjacent beach. Using the geoaccumulation index, we found that these two samples were moderately polluted with their *I*_geo_ being less than 2 [9], while all the other samples were assessed as relatively unpolluted. Therefore, since the prerequisite standards of the dredged material were met, the first planned opportunistic beach nourishment project in Greece materialized.

The frequent evaluation of the evolution of Platanias beach is continuing. About a year and a half later, it appears that opportunistic beach nourishment in specific locales (versus the entire beach) cannot solely address erosion, yet buys time, until a permanent and sustainable solution is implemented.

## 4. Conclusions

Our study (a) assessed the presence and distribution of heavy metals in coastal sediment from Chania beaches and ports, (b) identified heavy metal point sources in the coastal environment, and (c) assessed the feasibility of opportunistic beach nourishments projects in Greece. From the comparative assessment of the examined samples, we conclude the following.

- The majority of the examined beaches had similar heavy metal concentrations with background values (beachrock).
- Heavy metals point sources include municipal WWTP effluents, which led to increased loads of Zn in the 20 cm deep sediments.
- Likewise, a small tannery in Agia Kyriaki appears to have polluted the sediments an adjacent small port.
- Most of the examined sediments in ports and harbors exhibited low heavy metal concentrations in contrast to the higher loads on ports breakwaters, where substandard boat maintenance activities, such as repainting, often takes place. The source of the heavy metals is likely from pigments and dyes.

Opportunistic beach nourishment is an attractive method for temporarily alleviating anthropogenic beach erosion. Greece is among the countries with insufficient legislation concerning dredging activities, despite its extremely long coastline. Therefore, a reexamination of the current legislation towards more sustainable and environmentally friendly beach management may be advisable.

## Figures and Tables

**Table 1:** The sampling points, their coordinates, and a brief description.

*a*/*a *	Latitude	Longitude	Description
1	35.532731°	23.895444°	Surface sediment from the exit of Platanias WWTP at 12 m water depth.
2	35.532731°	23.895444°	Deep (20 cm) sediment from the exit of Platanias WWTP at 12 m water depth.
3	35.524739°	23.893374°	Surface sediment close to the delta of Platanias river, 5 m water depth.
4–7	35.519585°	23.911989°	Surface sediment from Platanias port.
8	35.513104°	23.951622°	Surface sediment from Kato Stalos beach, next to the exit of a small stream.
9	35.512890°	23.953652°	Surface sediment from the exit of a small stream in Kato Stalos beach.
10	35.513441°	23.964129°	Deep (20 cm) sediment from Kato Galatas Port.
11	35.513120°	23.963718°	Surface sediment from Kato Galatas Port.
12-13	35.512849°	23.964022°	Deep (20 cm) sediment from Kato Galatas Port.
14–17	35.512176°	24.001272°	Surface sediment from kladisos river delta.
18	35.515931°	24.007655°	Nea Chora port, sediment from the breakwater, where ship maintenance activities take place.
19	35.516331°	24.008479°	Surface sediment from Nea Chora port.
20–22	35.519784°	24.018881°	Surface sediment from old harbor of Chania.
23-24	35.516112°	24.029507°	Surface sediment from Koum Kapi beach, Chania.
25–28	35.519819°	24.037042°	Surface Sediment from Agia Kyriaki beach.
29	35.522146°	24.045043°	Sediment from the effluents of a small tannery in Agia Kyriaki bay.
30	35.523117°	24.046336°	Deep (20 cm) sediment from Agia Kyriaki port.
31	35.591935°	24.149655°	Surface sediment from the remote pocket beach of Agia Triada.
32	35.496533°	24.061187°	Surface sediment from Souda beach.
33	35.488996°	24.067110°	Surface sediment the exit of a small stream in Souda bay.
34	35.182981°	24.247641°	Surface sediment from Orthi Ammos beach, Frangokastello Sfakia.
35	35.200961°	24.136048°	Surface sediment from Sfakia beach, Sfakia.
36	35.201933°	24.109092°	Surface sediment from Glyka Nera beach, Sfakia.
37	35.199868°	24.078939°	Surface sediment from Loutro beach, Sfakia.
38	35.469402°	23.563673°	Mean value of sediment from 10 greenhouses, Falasarna beach, Kasteli.
39	35.470777°	23.564290°	Surface sediment from Falasarna beach, Kasteli.
40	35.477558°	23.568266°	Deep (20 cm) sediment from Falasarna port, Kasteli.
41	35.503813°	23.576891°	Surface sediment from Falasarna small beach, Kasteli.
